# Modifying reaction time tasks parameters in the automated IntelliCage identifies heightened impulsivity and impaired attention in the 3xTg-AD model of Alzheimer’s disease

**DOI:** 10.3389/fnagi.2024.1466415

**Published:** 2024-12-18

**Authors:** Jessica M. Judd, Wendy Winslow, Ian McDonough, Faizan Mistry, Ramon Velazquez

**Affiliations:** ^1^Arizona State University-Banner Neurodegenerative Disease Research Center at the Biodesign Institute, Arizona State University, Tempe, AZ, United States; ^2^School of Life Sciences, Arizona State University, Tempe, AZ, United States; ^3^Arizona Alzheimer’s Consortium, Phoenix, AZ, United States

**Keywords:** IntelliCage, reaction time task, 3xTg-AD mouse, Alzheimer’s disease, impulsivity, attention

## Abstract

**Background:**

The 3xTg-AD transgenic mouse model of Alzheimer’s disease (AD) is an important tool to investigate the relationship between development of pathological amyloid-β (Aβ) and tau, neuroinflammation, and cognitive impairments. Traditional behavioral tasks assessing aspects of learning and memory, such as mazes requiring spatial navigation, unfortunately suffer from several shortcomings, including the stress of human handling and not probing species-typical behavior. The automated IntelliCage system was developed to circumvent such issues by testing mice in a social environment while measuring multiple aspects of cognition. Water consumption can serve as a primary motivator for task engagement. Once animals adapt to the cage and can access water, mice can be subjected to operant tasks. Each of the four corners of a cage contains doors to manipulate access to water, visual LED cues, and a valve allowing administration of an air puff. Previously, we detected significant impairments in 3xTg-AD mice in the IntelliCage, however a high failure rate and genotypical differences in water motivation were observed.

**Methods:**

Here, we implemented an IntelliCage paradigm where mice underwent progressively more difficult reaction time tasks to assess attention and impulsivity, behaviors mediated by the prefrontal cortex. Mice were placed in the IntelliCage at 11.5 months of age, which corresponds with the presence of widespread pathology.

**Results:**

As the difficulty of the reaction time tasks increased, 3xTg-AD mice exhibited lower percent Correct Responses than NonTg. When implementing varying pre-cue durations, where animals are required to wait between the initiation of the trial and the LED turning on (which then requires a nose-poke to access water), 3xTg-AD mice prematurely nose-poked on trials requiring a longer delay before a second nose poke would allow water access, demonstrating heightened impulsivity. The presence of soluble and insoluble fractions of cortical Aβ40 and 42, and phosphorylated tau epitopes threonine 181 and serine 396 confirmed the presence of neuropathological hallmarks in 3xTg-AD mice.

**Conclusion:**

Together, this study describes a novel protocol that overcomes motivational differences and detects attention and impulsivity deficits in 3xTg-AD mice utilizing the IntelliCage.

## 1 Introduction

Alzheimer’s disease (AD) is an age-related neurodegenerative disease that currently afflicts 6.7 million people aged 65 and over in the United States (Alzheimer’s Association, 2024). The primary clinical manifestations of AD are progressive memory loss and executive dysfunction (Alzheimer’s Association, 2024; Scheltens et al., 2021). Eventually, AD leads to impairments in basic functions, such as swallowing, ultimately leading to death (Alzheimer’s Association, 2024; Scheltens et al., 2021). AD is characterized by the accumulation of amyloid beta (Aβ) plaques and neurofibrillary tau tangles (NFTs) (Alzheimer’s Association, 2024; Scheltens et al., 2021). While there are therapeutics that target Aβ pathology, they leave other aspects of the disease unattenuated (Alzheimer’s Association, 2024; Passeri et al., 2022). Thus, the need continues for further investigation into AD disease pathogenesis and pathology.

The triple transgenic mouse (3xTg-AD) is a commonly used rodent model of AD. This mouse contains familial human mutations for App (Swedish), Presenilin 1 knockin (PSEN1 M146V), and MAPT (P301L) that result in Aβ pathology starting in the frontal cortex at 6 months of age and elevations in pathological tau phosphorylation starting at 6 months in the hippocampus; pathology is widespread at 12 months of age (Oddo et al., 2003; Winslow et al., 2021). Additionally, these mice also display behavioral deficits that parallel AD’s clinical presentation. For example, learning and memory deficits can be detected as early as 6 months and become progressively worse by 12 months in spatial learning and memory tasks, such as the Morris Water Maze (MWM) (Parachikova et al., 2010; Roda et al., 2020; Winslow et al., 2021). This recapitulation of human disease manifestation has made the 3xTg-AD mouse an invaluable tool in studying the mechanisms behind AD and in preclinical assessment of the effectiveness of potential AD interventions and therapeutics.

A variety of behavioral assessments are well established in the neurodegenerative disease field and have aided research on the impact of AD pathogenesis and interventions, by providing insight into functional outcomes. Frequently used behavioral assessments tap into hippocampal dependent learning and memory and include spatial memory dependent mazes, such as the Morris Water Maze (MWM), the radial arm water maze (Commins and Kirby, 2019; Puzzo et al., 2014; Stewart et al., 2011; Tanila, 2012), the Barnes Maze (Harrison et al., 2006; Stewart et al., 2011), Y and T mazes (Stewart et al., 2011), and contextual fear conditioning (Ji and Maren, 2007; Maren et al., 2013). While these techniques have provided a wealth of information over the years, they possess experimental and logistical shortcomings. First, most tasks were originally designed for rats and then adapted to mice (Lipp et al., 2023; Tanila, 2012). For water maze testing, mice are more likely than rats to engage in behaviors that result in their exclusion from analysis, including floating and thigmotaxis (Tanila, 2012). Second, commonly used behavioral tests can be stressful for mice (Koolhaas et al., 2011), resulting in altered behavior compared to tests in a naturalistic setting (Lipp et al., 2023). Further, even when the behavioral assessment is not intrinsically stressful, mice do not acclimate to handling as well as rats and continue to be stressed by handling even after repeated exposures (Gouveia and Hurst, 2019; Lipp et al., 2023). Third, commonly used tasks are often unreflective of evolutionarily relevant challenges for mice (Lipp et al., 2023). Finally, traditional behavioral testing requires substantial experimental effort, with behavioral batteries or large cohorts often requiring weeks of experimenter labor (Lipp et al., 2023). Use of testing methods that overcome these limitations could lead to more productive research outcomes.

The IntelliCage was developed in 2000 by Dr. Hans-Peter Lipp and overcomes many of the traditional limitations encountered with mouse behavioral testing. The IntelliCage is a fully automated, operant system that allows rodents to be tested in a social environment (Dell’Omo et al., 2000; Lipp, 2005; Lipp et al., 2005). The IntelliCage has four operant corners where water access is used as the incentive for mice to participate in various experimental tasks. Within each corner, a mouse is identified by the detection of an implanted radio frequency identification and sensors for nose pokes and licks track these behaviors (Kiryk et al., 2020; Lipp et al., 2023; Masuda et al., 2018; Mifflin et al., 2021; Winslow et al., 2021). Using the IntelliCage, mice can be assessed for exploratory behavior, water consumption patterns, spatial learning, behavioral flexibility, attention, impulsivity, and working and contextual memory within a single cage and minimal experimental intervention. We and others have established that various mouse models of AD can be successfully tested in the IntelliCage (Codita et al., 2010; Masuda et al., 2018; Mifflin et al., 2021; Winslow et al., 2021). Recently, we found impairments on a reaction time tasks in 3xTg-AD mice in the IntelliCage compared to non-transgenic (NonTg) controls (Winslow et al., 2021). However, more complex and challenging operant reaction time task paradigms, with varying pre-cue delays and cue onsets, have only been minimally evaluated in the IntelliCage utilizing AD models.

In the present study, we tested NonTg and 3xTg-AD mice in the IntelliCage implementing variable pre-cue delays and cue onsets for the reaction time task, thereby requiring high demand of attention and suppression of impulsivity to complete. We made the reaction time tasks progressively more challenging, advancing from a task with fixed pre-cue durations, to tasks with both variable pre-cue and shorter cue-onset durations. Additionally, to better equalize motivation between genotypes, we limited water access to 3-h during the active phase of the light cycle, similar to that previously performed in another mouse model of AD (Masuda et al., 2016, 2018). We hypothesized that 3xTg-AD would display impaired attention and heightened impulsivity as the reaction time tasks became more challenging, compared to NonTg.

## 2 Methods

### 2.1 Animals

3xTg-AD mice were generated on a C57BL6/129Svj hybrid background as previously described (Dave et al., 2023; Judd et al., 2023; Oddo et al., 2003; Velazquez et al., 2019; Winslow et al., 2021). Since 3xTg-AD males show large neuropathological variability, only female mice were included in this study similar to previously published work (Dave et al., 2023; Judd et al., 2023; Velazquez et al., 2019; Winslow et al., 2021). All protocols were approved by the Institutional Animal Care and Use Committee of Arizona State University and conformed to the National Institutes of Health Guide for the Care and Use of Laboratory Animals. Mice were group housed (4 to 5 mice per cage) prior to being introduced into the IntelliCage. Prior to IntelliCage testing, a radiofrequency identification transponder chip (RFID; Standard Microchip T-VA, DataMars, Switzerland and Troven, United States) was subcutaneously implanted into the dorso-cervical region under isoflurane inhalation anesthesia as previously described (Mifflin et al., 2021; Winslow et al., 2021). The RFID chip allows for the identification of a mouse when it enters a corner of the IntelliCage system. Mice were allowed at least 2 weeks to recover from transponder implantation and were then introduced into the IntelliCage. IntelliCage testing started at 11.5 months (range 10.7–12.5 months), when amyloidosis is advanced, tau pathology is prevalent in the hippocampus, and cognitive impairments are observed (Dave et al., 2023; Judd et al., 2023; Oddo et al., 2003; Velazquez et al., 2019; Winslow et al., 2021).

Female 3xTg-AD (n = 10) and C57BL6/129Svj non-transgenic controls (n = 14; NonTg) mice were placed in the IntelliCage for assessment across a variety of tasks that tapped into hippocampal and prefrontal cortical function (Ajonijebu et al., 2018; Kiryk et al., 2020; Voikar et al., 2018). IntelliCage testing took a total of 34 days from Adaptation to the Place Avoidance retention phase (detailed below). Each IntelliCage holds up to 16 mice, and our lab has four cages. Two NonTg mice were excluded from testing following the Reversal task for failure to engage in the task. Mice were euthanized 2 weeks after the conclusion of testing, and tissue was dissected and prepared for ELISAs analysis to assess pathogenesis.

### 2.2 Behavioral testing

We have utilized the IntelliCage as described previously (Winslow et al., 2021). The IntelliCage testing apparatus (39 cm × 58 cm × 21 cm) contains four operant corner chambers which are accessible through an antenna-equipped open tunnel. The IntelliCage is filled with Diamond Dry bedding. To avoid disturbing the mice, bedding was not changed during the duration of testing. A computer program regulates water access through two individual doors in each corner. At each corner entrance, a scanner registers an animal’s presence by scanning the mouse’s RFID. For an RFID to be read and a corner visit to be counted, an animal’s entire body must enter the corner. Nose pokes and licks are detected by sensors on the nose port and waterspout, respectively. Mice had *ad libitum* access to standard rodent chow throughout IntelliCage testing. Lights were on in the behavior room from 06:00 to 18:00. Video cameras were placed outside of the IntelliCages and recorded the entire testing sessions. The sequence of experimental behavioral tasks in the IntelliCage was as follows: (1) Adaptation, consisting of Free Adaptation, Door Adaptation, and Nose Poke Adaptation phases; (2) Place Preference and Reversal; (3) Reaction Time Task with varying pre-cue delays and cue onsets; and (4) Place Avoidance and Extinction. Any animal that failed to consume water in a 48-h period were removed from the IntelliCage and placed in a standard cage with accessible water for 7-h to avoid severe dehydration. If these animals were re-introduced into the IntelliCage and again failed to consume water, they were removed from the experiment.

Data were extracted using the TSE IntelliCagePlus Analyzer software, exported into multiple tab-delimited text files, and then, using a Python script, converted into a single SQLite3 database file as previously described (Winslow et al., 2021). Using this file as input, several Python scripts were then used to query the database using SQL to extract relevant dependent variables. The same script also sliced the data into 24-h periods and separated the data into Excel spreadsheets with the data for each day. For each task, the dependent variables calculated are described below.

#### 2.2.1 Adaptation phases

**Figure 1:**
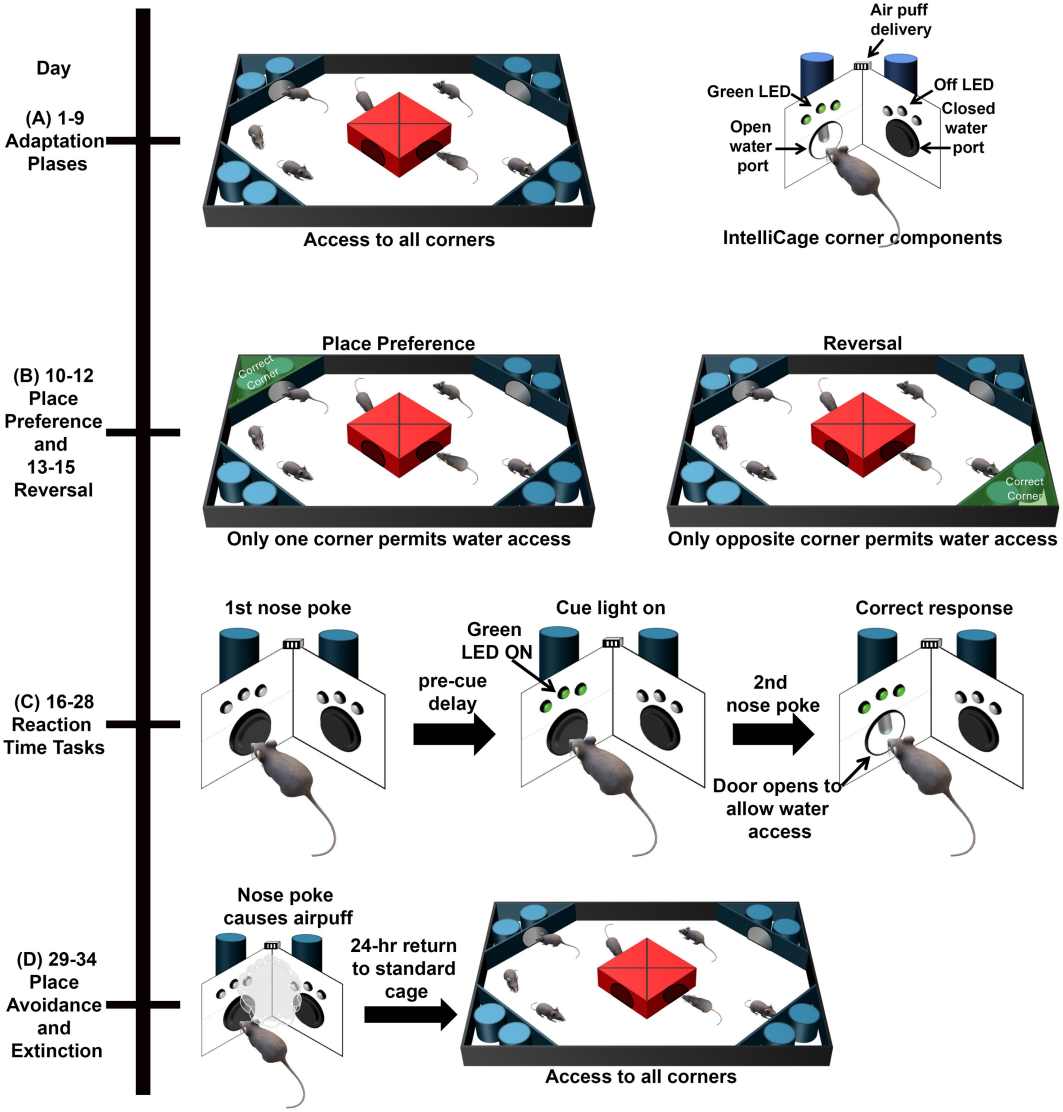
Schematic of the IntelliCage tests. **(A)** During the adaptation phases, mice were acclimated to the IntelliCage and the procedure of nose poking to get the door to open for water access. **(B)** During Place Preference, water was only accessible from one corner. During Reversal, water was only available from the opposite corner. **(C)** During Reaction Time Tasks, mice initiated a trial with a nose poke at the assigned corner. Mice then had to wait during the pre-cue delay before an LED is illuminated to perform a second nose poke and access water. **(D)** During Avoidance, mice encountered an air puff when they entered the assigned corner and nose poked. After being placed in a standard cage for 24-h, mice were returned to the IntelliCage for Extinction and Retention with water access from all corners.

The Adaptation phases (Figure 1A) consisted of a variety of shaping behaviors to acclimate the mice first to the IntelliCage, then to the procedure of nose poking at a corner to open the doors, allowing water access, and finally to the restricted water access timeframe.

##### 2.2.1.1 Free Adaptation (days 1–4)

During Free Adaptation, all doors were open to allow free access to the water bottles to acclimate mice to the new environment. No water restriction period was implemented.

The following were calculated for the Free Adaptation phase:

- Total Visits = The total number of all corner visits. This is a measure of exploratory and water-seeking behavior.
- Total Licks = The total number of licks made on a given day. This is a measure of water consumption.

##### 2.2.1.2 Door Adaptation (days 5–6)

During Door Adaptation, the doors to the water bottles were closed, but they opened for any visit into the corner. No water restriction period was implemented. Total Visits and Total Licks were measured.

##### 2.2.1.3 Nose Poke Adaptation (days 7–8)

For Nose Poke Adaptation, animals were trained to nose poke to initiate water access. Doors were closed and would open following a nose poke in a corner. No water restriction period was implemented. In addition to Total Visits and Total Licks, the following outcomes for this task were:

- Total Visits with Nose Poke = Number of visits with one or more nose poke, which indicated successful shaping to the task.
- Visits with ≥1 Lick = Number of visits with one or more licks, which indicates the number of visits with clear water seeking behavior.

##### 2.2.1.4 Water Deprivation Adaptation (day 9)

Genotypic differences in water seeking behavior in the IntelliCage can influence performance (Winslow et al., 2021). To equalize motivation, in the next phase of Adaptation, we implemented water restriction, similar to a publication in that used another rodent model of AD (Masuda et al., 2016, 2018). Starting with this Adaptation phase, mice were only able to access water during three of the 12-h dark phase, when mice are more active. In addition to the metrics analyzed during Nose Poke Adaptation, the following metric was also assessed:

- Total Visits During Water Access = The number of visits made during the 3-h specifically when water could be accessed.

#### 2.2.2 Place Preference (days 10–12) and Reversal (days 13–15)

During the Place Preference (PP) phase (Figure 1B), water was accessible in only one of the four corners for each of the mice. For each mouse, their least visited corner from the Adaptation phases was selected as the assigned corner to eliminate potential preferential corner bias. During Reversal, water was available only in the opposite corner from PP. The rewarded corners were balanced by the number of mice and genotype, to prevent overcrowding of the corners and learning by imitation as previously described (Winslow et al., 2021). In addition to measuring Total Visits and Total Licks per day, outcomes for this task included:

- Assigned Visits = Number of visits to the assigned corner
- Assigned Visits with ≥1 Lick = Number of visits to the assigned corner that included at least one lick
- %Correct Response = (Assigned Visits with ≥ 1 Lick/Total Visits) *100

#### 2.2.3 Reaction Time (RT) attention Task

During the Reaction Time (RT) Task (Figure 1C), when a mouse entered an assigned corner, which was the same corner as assigned in the Reversal PP, the first nose poke of a visit initiated a trial. Then, a pre-cue delay was imposed prior to the illumination of a green LED, which required the mice to learn to wait for the green LED before proceeding, assessing impulsivity. While the green LED was on, mice could nose poke again, thereby assessing attention, which would result in the door opening and permitting water access. Any nose poke before or after the green LED was illuminated resulted in an error and the mouse had to initiate a new trial to attempt to access water. By manipulating the pre-cue delay and the time of cue onset, RT tasks progressively increased in difficulty for animals. The RT sessions had the following permutations:

- Days 16–18: A 2 s pre-cue followed by 7 s with the green LED on.
- Days 19–21: A variable 2, 4, or 8 s pre-cue followed by 7 s with the green LED on.
- Days 22–24: A variable 2, 4, or 8 s pre-cue followed by 3 s with the green LED on.
- Days 25–28: A variable 2, 4, or 8 s pre-cue followed by 1 s with the green LED on.

We measured the following three outcomes for the initiated trials:

- Abandoned Trials = Trials without a second nose poke during the cue duration.
- Premature Response = Trials with a nose poke during the pre-cue delay, before the green LED comes on. This is a measure of impulsivity.
- Correct Response = Trials with a second nose poke during the time when the green LED was on.

As in previous sessions, Total Visits and Total Licks were measured. The following measures were also reported:

- Trials = The number of initiated trials.
- %Abandoned Trials = (Total Abandoned Trials/Total Trials) * 100
- %Premature Responses = (Total Trials with Premature Response/Total Trials) * 100
- %Correct Responses = (Total Trials with Correct Response/Total Trials) * 100

Additionally, for the sessions with a variable pre-cue duration, these metrics were broken down by the individual pre-cue durations (ex. %Abandoned Trials with 2 s pre-cue = (Abandoned Trials with 2 s pre-cue/Trials with 2 s pre-cue) * 100).

#### 2.2.4 Place Avoidance (days 29–34)

The Place Avoidance task (Figure 1D) taps contextual memory mediated by the hippocampus, and includes both training (learning) and probe (memory and extinction) trials. For the one day, 24-h training trial (avoidance training), a nose poke in the reward corner resulted in an air puff (~0.8 bar, 1 s air-puff). The doors in all corners remained closed and water was not available during the learned avoidance phase. In addition to measuring total visits per day, we also analyzed the number of corner visits with nose pokes at the air puff corner to assess working memory errors.

After the 24-h training trial, the mice were moved to their standard home cages for a 24-h delay with water *ad libitum*. After the delay, the mice were reintroduced to the IntelliCage for 4 days with water available at all four corners and the air puff stimulus removed to assess retention and extinction.

### 2.3 Sacrifice and tissue collection

Following IntelliCage testing, mice were sacrificed via perfusion with 1 × Phosphate buffer saline (PBS) and brains were harvested. One hemisphere had the cortex dissected out, flash-frozen with dry ice, and stored at −80°C. Extracted tissue was homogenized in a T-PER tissue protein extraction reagent supplemented with protease (Roche Applied Science, IN, United States) and phosphatase inhibitors (Millipore, MA, USA). The homogenized tissues were centrifuged at 4°C for 30 min, and the supernatant (soluble fraction) was stored at −80°C. The remaining pellet was further homogenized in 70% formic acid and then centrifuged at 4°C for 30 min. The supernatant (insoluble fraction) was collected, neutralized, and stored at −80°C.

### 2.4 ELISAs

To validate amyloid and tau pathology in the 3xTg-AD mice, commercially available ELISA kits (Invitrogen-ThermoFisher Scientific) were used to quantify cortical levels of soluble and insoluble Aβ40 (Cat# KHB3481) and Aβ42 (Cat# KHB3441), and levels of phosphorylated Tau (pTau) at threonine (T) 181 (Cat# KHO0631) and serine (S) 396 (Cat# KHB7031) as previously described (Dave et al., 2023; Judd et al., 2023; Winslow et al., 2021).

### 2.5 Statistical analysis

Repeated measures Analysis of Variance (ANOVA) test were performed in SPSS (IBM, v28.0.1.1) to analyze behavior outputs across multiple days of testing. Data visualization, One-way ANOVA and students’ unpaired *t*-tests were performed in GraphPad Prism (v8.1.2). Bonferroni’s corrected *post hoc* tests were performed when a significant interaction was observed. Examination of descriptive statistics revealed no violations of any assumptions that required the use of statistical tests other than the ones used. Output for significant ANOVAs and *t*-test are displayed in Table 1. Significance was set at *p* < 0.05.

**Table 1:** Statistical output of significant ANOVAs and *t*-tests for the IntelliCage tests.

Dependent variable	ME Geno		ME Day		Geno × Day	
	*F*	*p*	*F*	*p*	*F*	*p*
***Adaptations***						
*Free Adaptation*						
Total Visits			*F*(3,45) = 4.532	*p* = 0.007	*F*(3,45) = 4.999	*p* = 0.004
Total Licks			*F*(3,45) = 4.577	*p* = 0.007	*F*(3,45) = 3.071	*p* = 0.037
*Door Adaptation*						
Total Visits						
Total Licks	*F*(1,22) = 5.019	*p* = 0.035	*F*(1,22) = 5.032	*p* = 0.035		
*Nose Poke Adaptation*						
Total Visits	*F*(1,22) = 7.826	*p* = 0.010				
Total Licks			*F*(1,22) = 4.396	*p* = 0.048		
Visits with a Nose Poke	*F*(1,22) = 4.853	*p* = 0.038				
Visits with ≥1 Lick	*F*(1,22) = 7.088	*p* = 0.014				

Dependent variable	ME Geno	
	*t*	*p*
*Water Restriction Adaptation*		
Total Visits		
Total Licks		
Visits with a Nose Poke		
Visits with ≥1 Lick		
Visits During Water Access	*t*(22) = 2.158	*p* = 0.042

Dependent variable	ME Geno		ME Day		Geno × Day	
	*F*	*p*	*F*	*p*	*F*	*p*
***Place Preference and Reversal***						
*Place Preference*						
Total Visits					*F*(2,44) = 3.157	*p* = 0.052
Total Licks			*F*(2,44) = 4.351	*p* = 0.019		
Assigned Visits						
Assigned Visits with ≥1 Lick	*F*(1,22) = 4.241	*p* = 0.051	*F*(2,44) = 5.576	*p* = 0.007		
%Correct Responses			*F*(2,44) = 7.913	*p* = 0.001		
*Reversal*						
Total Visits						
Total Licks						
Assigned Visits						
Assigned Visits with ≥1 Lick	*F*(1,22) = 7.229	*p* = 0.013				
%Correct Responses			*F*(2,44) = 5.133	*p* = 0.010		
						
***Reaction Time Task***						
*Reaction Time Task 2 s pre-cue with 7 s cue*						
Total Visits			*F*(2,38) = 3.147	*p* = 0.054		
Total Licks			*F*(2,38) = 3.343	*p* = 0.046		
Total Initiated Trials						
%Abandoned Trials			*F*(2,38) = 21.774	*p* < 0.001		
%Premature Response						
%Correct Responses			*F*(2,38) = 4.736	*p* = 0.015		
*Reaction time task variable 2, 4, or 8 s pre-cue with 7 s cue*						
Total Visits	*F*(1,19) = 5.225	*p* = 0.034				
Total Licks	*F*(1,19) = 5.089	*p* = 0.036				
Total Initiated Trials	*F*(1,19) = 5.810	*p* = 0.026				
%Abandoned Trials (All Trials)						
%Abandoned Trials (Trials with 2 s pre-cue)						
%Abandoned Trials (Trials with 4 s pre-cue)						
%Abandoned Trials (Trials with 8 s pre-cue)						
%Premature Responses (All Trials)			*F*(2,38) = 5.354	*p* = 0.009		
%Premature Responses (Trials with 2 s pre-cue)						
%Premature Responses (Trials with 4 s pre-cue)			*F*(2,38) = 4.277	*p* = 0.021		
%Premature Responses (Trials with 8 s pre-cue)						
%Correct Responses (All Trials)			*F*(2,38) = 11.265	*p* < 0.001		
%Correct Responses (Trials with 2 s pre-cue)			*F*(2,38) = 4.364	*p* = 0.020		
%Correct Responses (Trials with 4 s pre-cue)			*F*(2,38) = 6.067	*p* = 0.005		
%Correct Responses (Trials with 8 s pre-cue)			*F*(2, 38) = 4.065	*p* = 0.025		
*Reaction Time Task variable 2, 4, or 8 s pre-cue with 3 s cue*						
Total Visits	*F*(1,19) = 7.63	*p* = 0.012				
Total Licks	*F*(1,19) = 10.502	*p* = 0.004				
Total Initiated Trials	*F*(1,19) = 13.956	*p* = 0.001			*F*(2, 38) = 3.539	*p* = 0.039
%Abandoned Trials (All Trials)						
%Abandoned Trials (Trials with 2 s pre-cue)						
%Abandoned Trials (Trials with 4 s pre-cue)						
%Abandoned Trials (Trials with 8 s pre-cue)						
%Premature Responses (All Trials)						
%Premature Responses (Trials with 2 s pre-cue)						
%Premature Responses (Trials with 4 s pre-cue)	*F*(1,19) = 4.172	*p* = 0.055				
%Premature Responses (Trials with 8 s pre-cue)						
%Correct Responses (All Trials)						
%Correct Responses (Trials with 2 s pre-cue)						
%Correct Responses (Trials with 4 s pre-cue)	*F*(1,19) = 4.508	*p* = 0.047				
%Correct Responses (Trials with 8 s pre-cue)			*F*(2,38) = 3.504	*p* = 0.040		
*Reaction Time Task variable 2, 4, or 8 s pre-cue with 1 s cue*						
Total Visits	*F*(1,19) = 9.112	*p* = 0.007	*F*(3,57) = 12.405	*p* < 0.001	*F*(3,57) = 3.117	*p* = 0.033
Total Licks	*F*(1,19) = 7.120	*p* = 0.015				
Total Initiated Trials	*F*(1,19) = 15.186	*p* < 0.001				
%Abandoned Trials (All Trials)						
%Abandoned Trials (Trials with 2 s pre-cue)						
%Abandoned Trials (Trials with 4 s pre-cue)						
%Abandoned Trials (Trials with 8 s pre-cue)						
%Premature Responses (All Trials)						
%Premature Responses (Trials with 2 s pre-cue)						
%Premature Responses (Trials with 4 s pre-cue)						
%Premature Responses (Trials with 8 s pre-cue)						
%Correct Responses (All Trials)	*F*(1,19) = 6.556	*p* = 0.019				
%Correct Responses (Trials with 2 s pre-cue)						
%Correct Responses (Trials with 4 s pre-cue)			*F*(3,57) = 5.388	*p* = 0.002		
%Correct Responses (Trials with 8 s pre-cue)	*F*(1,19) = 9.682	*p* = 0.006				

Dependent variable	ME Geno	
	*t*	*p*
***Place Avoidance, and Retention and Extinction***		
*Place Avoidance*		
Total Visits	*t*(19) = 2.575	*p* = 0.0186
Assigned Visits		
Assigned Visits with Nose Poke		

Dependent variable	ME Geno		ME Day		Geno × Day	
	*F*	*p*	*F*	*p*	*F*	*p*
*Retention and Extinction*						
Total Visits			*F*(3,57) = 5.902	*p* = 0.001		
Total Licks			*F*(3,57) = 4.620	*p* = 0.006		
Assigned Visits with Nose Poke			*F*(3,57) = 3.717	*p* = 0.016		

## 3 Results

### 3.1 Both 3xTg-AD and NonTg mice completed all adaptation phases of the IntelliCage, with 3xTg-AD exhibiting increased water seeking behavior

**Figure 2:**
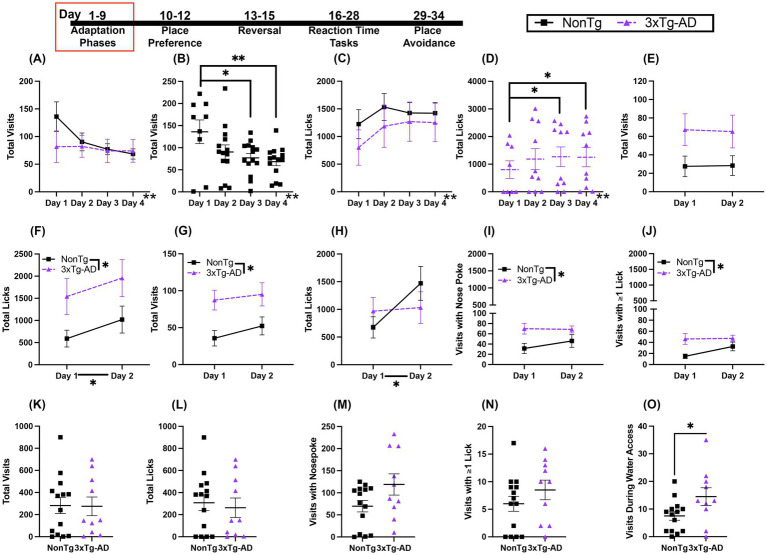
Both genotypes acquired the rules of the IntelliCage tasks. **(A)** Across the Free Adaptation days, the number of Total Visits decreased and **(B)** NonTg mice made fewer visits on days 3 and 4 than on day 1. **(C)** Total Licks increased across Free Adaptation days and **(D)** 3xTg-AD mice made more licks on days 3 and 4 than on day 1. **(E)** During Door Adaptation, there were no significant difference in Total Visits, but **(F)** 3xTg-AD mice made more Total Licks than NonTg and there were more Total Licks on day 2 than day 1. **(G)** 3xTg-AD mice made more Total Visits during Nose Poke Adaptation than NonTg. **(H)** Total Licks during Nose Poke Adaptation increased across days. During Nose Poke Adaptation, 3xTg-AD mice made more **(I)** Visits with a Nose Poke and **(J)** Visits with ≥1 Lick than NonTg. During the Water Restriction Adaptation, **(K)** Total Visits, **(L)** Total Licks, **(M)** Visits with Nose Poke, and **(N)** Visits with ≥1 Lick were similar between groups, but **(O)** 3xTg-AD made more Visits During Water Access than NonTg.

Across the Free Adaptation training days, we found that the number of Total Visits significantly decreased (Figure 2A). Additionally, we found a significant day by genotype interaction (Figure 2B), where the NonTg mice made fewer visits on days 3 (*p* = 0.014) and 4 (*p* = 0.005) than on day 1. 3xTg-AD did not significantly differ in their number of visits across days. When assessing Total Licks, we found a significant increase across Free Adaptation days (Figure 2C). There were more licks on day 2 than on day 1 (*p* = 0.044). A significant day by genotype interaction (Figure 2D) revealed that 3xTg-AD mice made more licks on days 3 (*p* = 0.029) and 4 (*p* = 0.020) than on day 1.

Next, mice were acclimated to the door mechanisms for corner access. Total Visits were similar across days and between genotypes (Figure 2E). For Total licks (Figure 2F), there were significantly more licks made by 3xTg-AD than NonTg across the 2 days. Further, there was an increase in licks from day 1 to day 2. Lastly, mice underwent Nose Poke Adaptation to learn to nose poke for the doors to open. For Total Visits (Figure 2G), 3xTg-AD mice made more visits than NonTg For Total Licks (Figure 2H), there was a significant effect of day, with more licks on day 2 than on day 1. There were significantly more Visits with a Nose Poke (Figure 2I) by 3xTg-AD than by NonTg. There were also significantly more Visits with ≥1 Lick (Figure 2J) by 3xTg-AD than by NonTg, illustrating 3xTg-AD made more visits for water access as opposed to exploration. Collectively, these results highlight that all mice were successfully shaped to the basic behavioral sequence required for water acquisition in the IntelliCage.

In the final phase of Adaption, water access was restricted and was available for only three hours during the active part of the light cycle (lights off). Over the entire Water Restriction Adaptation phase, there were no significant main effects or interactions for Total Visits, Total Licks, Visits with Nose Poke, or Visits with ≥1 Lick (Figures 2K–N). There was a significant genotype effect for Visits During Water Access (Figure 2O), with more visits made by 3xTg-AD mice than NonTg mice. These results highlight that both genotypes were able to successfully learn the procedure of the IntelliCage and complete Adaptation phases, with the 3xTg-AD mice making more visits and licks than NonTg at various stages of Adaption, reflecting higher water seeking behavior (Winslow et al., 2021).

### 3.2 NonTg and 3xTg-AD mice showed similar acquisition of both the Place Preference and Reversal learning tasks

**Figure 3:**
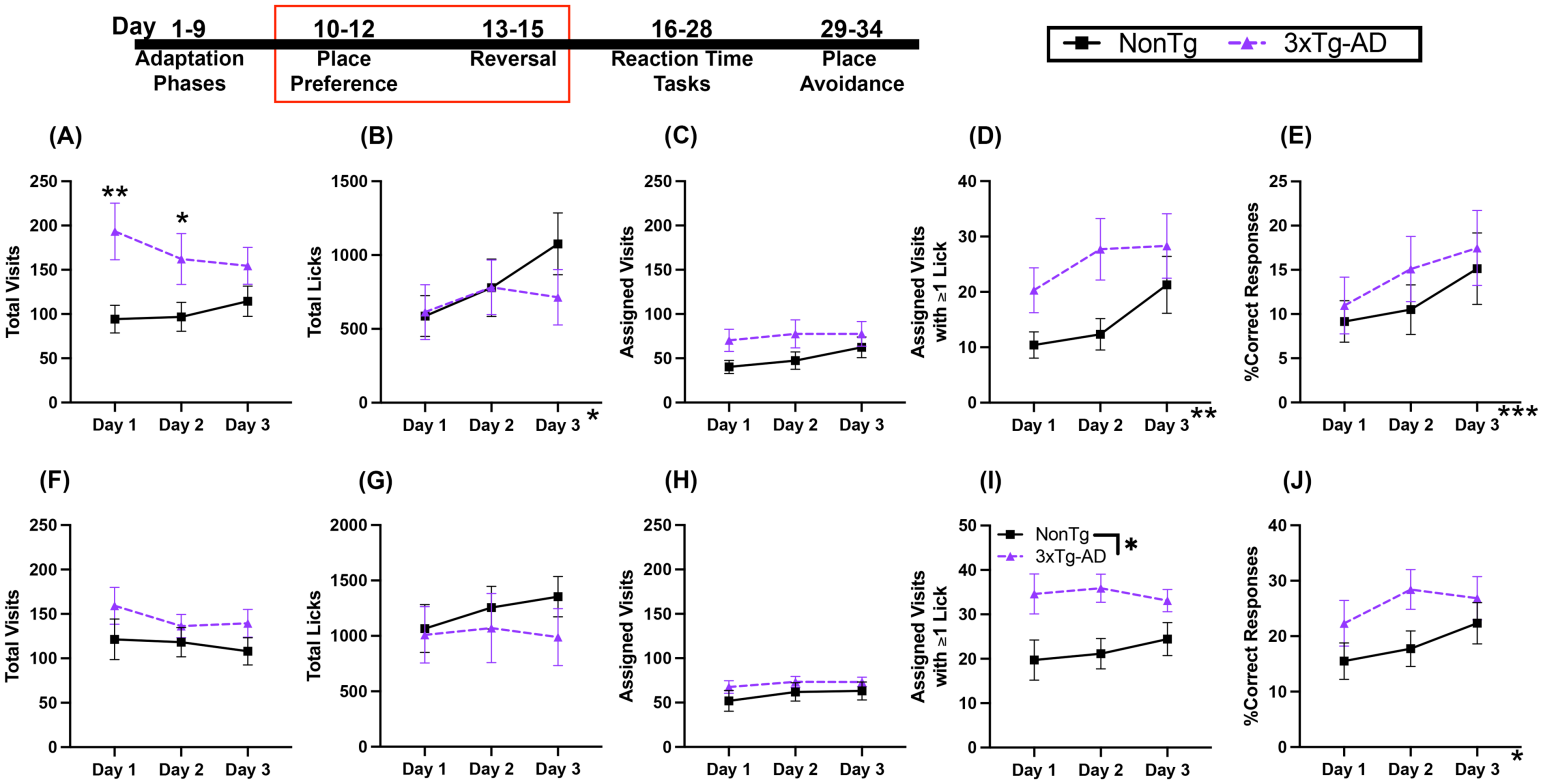
Both genotypes of mice learned to preferentially visit assigned corners. **(A)** During Place Preference (PP) 3xTg-AD made more Total Visits than NonTg on days 1 and 2. **(B)** Total Licks during PP increased across days. **(C)** While Assigned Visits were not different between genotypes or across days, **(D)** Assigned Visits with ≥1 Lick increased across days of PP. **(E)** PP %Correct Responses increased across days. For Reversal, there were no genotype differences or changes across days in **(F)** Total Visits, **(G)** Total Licks, or **(H)** Assigned Visits. **(I)** 3xTg-AD made more Assigned Visits with ≥1 Lick during Reversal than NonTg. **(J)** Reversal %Correct Response increased across days. Data are reported as means ± SEM. *p < 0.05, **p < 0.01, ***p < 0.001.

In the Place Preference (PP) task, mice can only access water from one assigned corner. For Total Visits during PP (Figure 3A), there was a trending interaction of day by genotype, with 3xTg-AD having more Total Visits than NonTg on day 1 (*p* = 0.006) and day 2 (*p* = 0.046). For Total Licks (Figure 3B), there was a significant effect of day, with more licks on day 2 (*p* = 0.023) and day 3 (*p* = 0.050) than on day 1. For number of Assigned Visits (Figure 3C), there were no significant main effects or interactions. For Assigned Visits with ≥1 Lick (Figure 3D), there was a significant day effect, with more Assigned Visits with ≥1 Lick on day 2 (*p* = 0.014) and day 3 (*p* = 0.035) than on day 1. Additionally, there was a trending genotype effect, with more Assigned Visits with ≥1 Lick by 3xTg-AD than NonTg. For %Correct Responses (Figure 3E), there was a day effect, with significantly higher %Correct Responses on day 3 than day 1 (*p* = 0.020) and a trend of higher %Correct Responses on day 3 than day 2 (*p* = 0.054).

In the Reversal PP task, animals can only access water from the corner opposite to the assigned corner in the PP task. For Total Visits, Total Licks, and Assigned Visits (Figures 3F–H), there were no significant main effects or interactions. For Assigned Visits with ≥1 Lick (Figure 3I), there was a significant genotype effect, where 3xTg-AD made more Assigned Visits with ≥1 Lick than NonTg. For %Correct Responses (Figure 3J), there was a day effect, but follow-up pairwise comparisons did not reveal significant differences between days. Both genotypes learned to preferentially visit the correct corner in both PP and Reversal PP. Additionally, 3xTg-AD continued to show higher motivation for water, with more Assigned Visits with ≥1 Lick than NonTg. Taken together, these results show that mice were able to similarly learn the correct corner for water access for both the initial PP task and the Reversal PP task.

### 3.3 Both genotypes show increased correct responses across days in the reaction time (RT) task with a fixed 2 pre-cue and a 7 s cue onset

**Figure 4:**
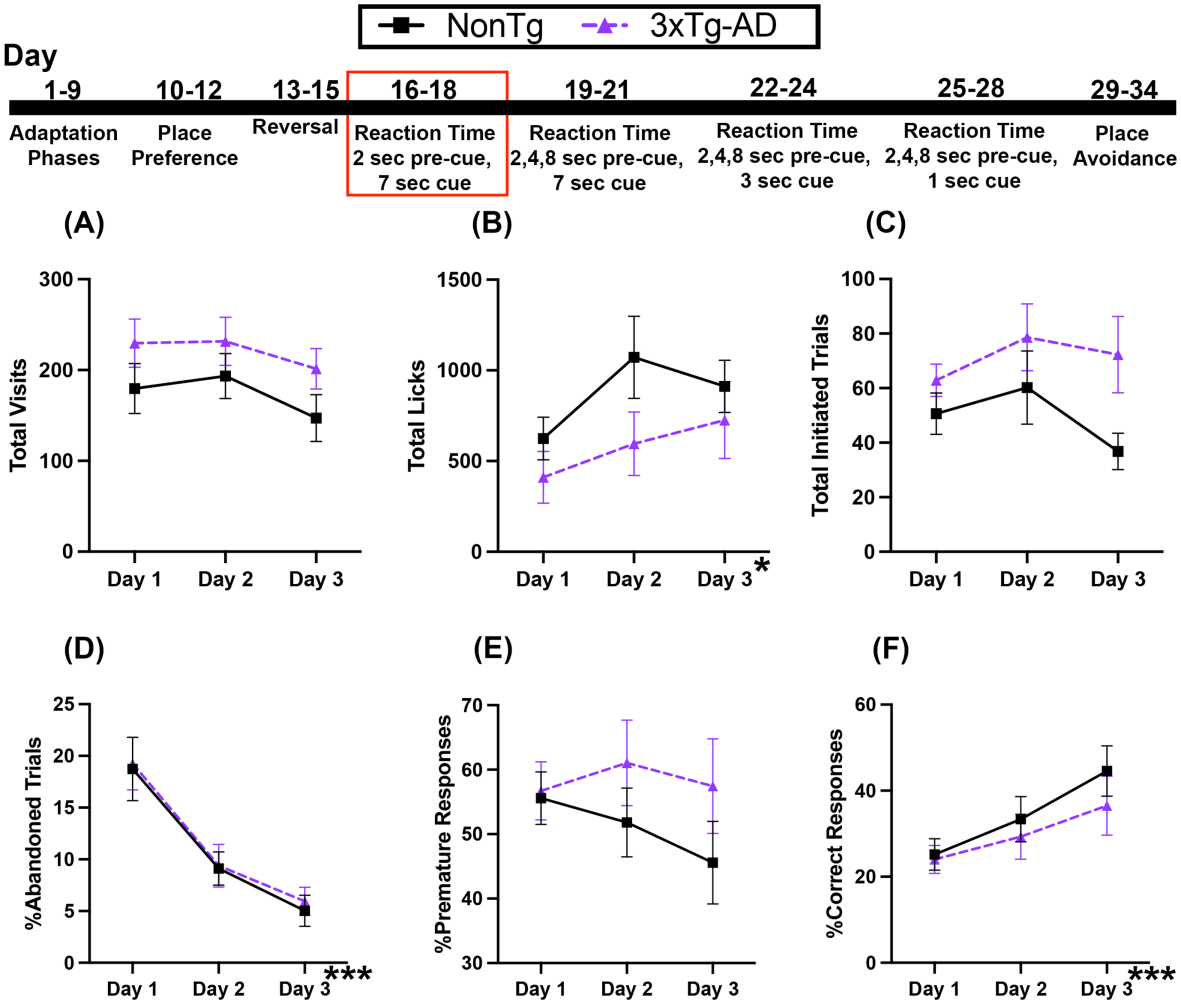
Both 3xTg-AD and NonTg mice showed improved performance across the 3-day 2 s fixed pre-cue and 7 s cue onset RT session. **(A)** No differences in Total Visits but **(B)** Total Licks increased for both genotypes across days. **(C)** Total Initiated Trials did not differ between genotype or days. **(D)** %Abandoned Responses decreased across days in both genotypes. **(E)** %Premature Response was not different between genotypes. Both groups showed an increase in **(F)** %Correct Response across days. Data are reported as means ± SEM. *p < 0.05, ***p < 0.001.

In this Reaction Time (RT) Task, the first nose poke at the assigned corner initiates a trial. Animals must learn to wait 2 s (pre-cue delay) before an LED is illuminated (and stays on for 7 s) to perform a second nose poke, extinguishing the LED, allowing access to water. For Total Visits (Figure 4A), there was a trending main effect of day, which suggested that visits decreased across days. For Total Licks (Figure 4B), there was a significant day effect, where licks increased across days. Total Initiated Trials (Figure 4C) were not significantly different across days or between the genotypes.

Next, we looked at responses during the task. The %Abandoned Responses (Figure 4D) decreased across days; the percent of trials that were abandoned on days 2 (*p* = 0.003) and 3 (*p* < 0.001) was less than on day 1, suggesting that both genotypes were able to learn the rules of the task across days. There were no significant main effects or interactions for %Premature Responses (Figure 4E), suggesting that the ability to wait during the pre-cue delay was similar on this task. For %Correct Response (Figure 4F), there was a significant effect of day, with higher percent correct on day 3 than day 1 (*p* = 0.035) regardless of genotype, indicated that all mice were able to acquire the rules of the task across time. Collectively, the increase in %Correct Response and the corresponding decrease in %Abandoned Responses across days suggests that both genotypes were able to similarly learn the rules of this RT task with only one pre-cue delay time of 2 s.

### 3.4 Varying the pre-cue between 2, 4, or 8 s with a 7 s cue onset reveals genotype differences

**Figure 5:**
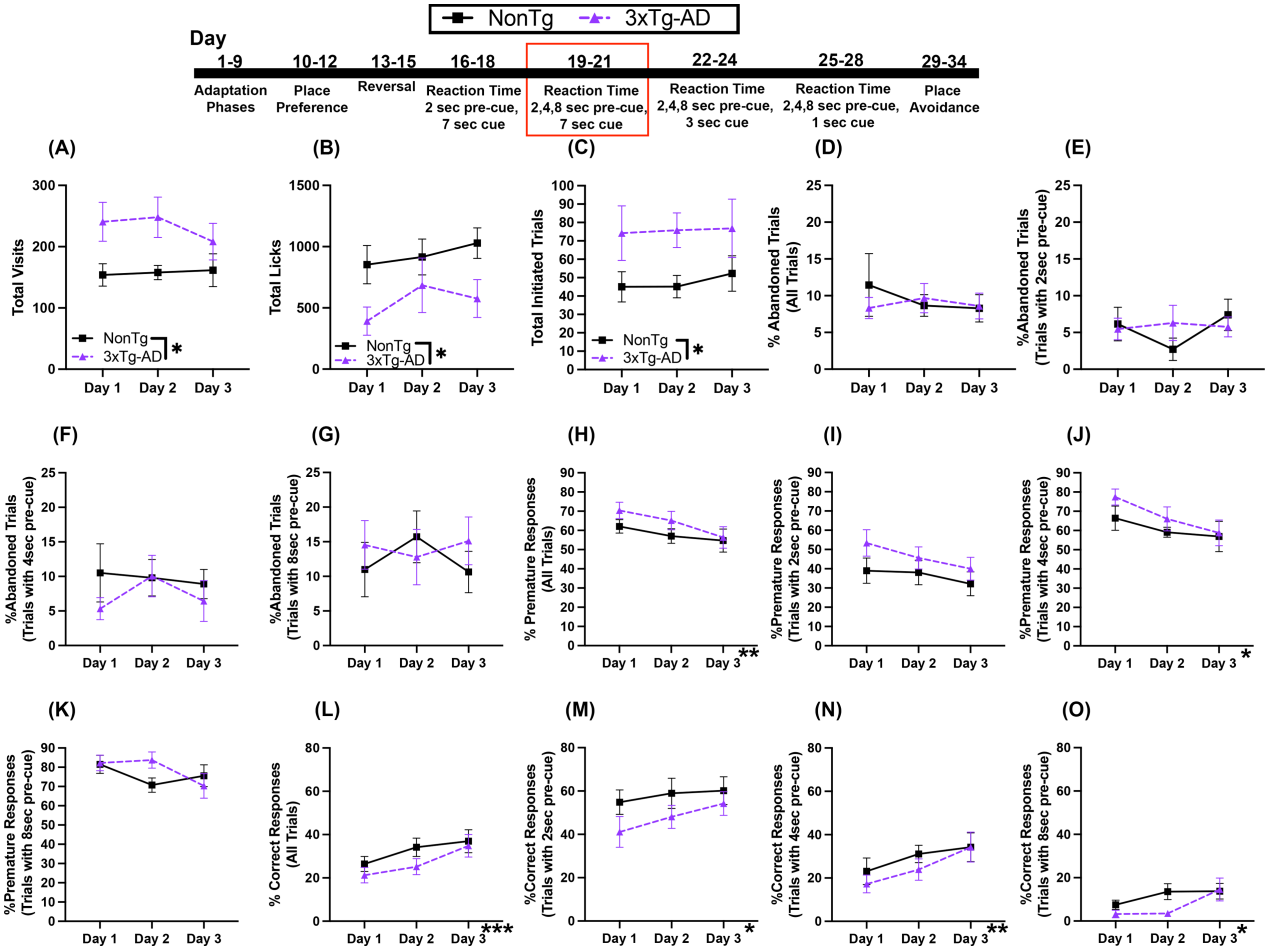
During the variable 2, 4, or 8 s pre-cue and 7 s cue duration RT task, 3xTg-AD mice initiated more trials, but %Correct Response was similar between groups and increased across days. **(A)** 3xTg-AD made more Total Visits than NonTg, but **(B)** NonTg made more Total Licks. 3xTg-AD initiated more **(C)** Total Trials. There were no significant differences for **(D)** %Abandoned Trials for all trials, **(E)** %Abandoned Trials with 2 s pre-cue, **(F)** %Abandoned Trials with 4 s pre-cue, or **(G)** %Abandoned Trials with 8 s pre-cue. There was a decrease across days in **(H)** %Premature Responses for all trials, **(I)** %Premature Responses for trials with 2 s pre-cue and **(J)** %Premature Responses for trials with 4 s pre-cues, but not for **(K)** %Premature Responses for trials with 8 s pre-cue. There was an increase across days for **(L)** %Correct Response for all trials, **(M)** %Correct Response for trials with 2 s pre-cue, **(N)** %Correct Response for trials with a 4 s pre-cue, and **(O)** %Correct Response for trials with an 8 s pre-cue. Data are reported as means ± SEM. *p < 0.05, **p < 0.01, ***p < 0.001.

In this RT Task, the first nose poke at the assigned corner initiates a trial in which the mouse must wait 2, 4, or 8 s before making a second nose poke to access water during the 7 s that the LED light cue is on. We found that there were more Total Visits (Figure 5A) by 3xTg-AD than NonTg. However, there were more Total Licks (Figure 5B) by NonTg than by 3xTg-AD. Additionally, 3xTg-AD mice initiated more Total Trials (Figure 5C).

There were no significant main effects or interactions for %Abandoned Trials for all trials (Figure 5D), %Abandoned Trials with 2 s pre-cue (Figure 5E), %Abandoned Trials with 4 s pre-cue (Figure 5F), or %Abandoned Trials with 8 s pre-cue (Figure 5G). Next, %Premature Responses for all trials (Figure 5H) decreased across days; with a lower %Premature Response on day 3 than day 1 (*p* = 0.034). While there were no significant main effects or interactions for %Premature Response for trials with a 2 s pre-cue (Figure 5I), %Premature Response on trials with 4 s pre-cue (Figure 5J) decreased across days, with lower %Premature Responses on day 3 than day 1 (*p* = 0.038). On trials with an 8 s pre-cue, there were no significant main effects or interactions in %Premature Responses (Figure 5K). Conversely, the total %Correct Response (Figure 5L), increased across days, with a higher %Correct Response on days 2 (*p* = 0.008) and 3 (*p* = 0.001) than on day 1. The increase in %Correct Response across days was also observed for the individual pre-cue trial durations. For trials with a 2 s pre-cue (Figure 5M), the %Correct Response increased across days, with significantly higher %Correct Responses on day 3 than day 1 (*p* = 0.043). For %Correct Response on trials with a 4 s pre-cue (Figure 5N), a significant day effect showed that there was a higher %Correct Response on day 3 than on day 1 (*p* = 0.008). For %Correct Response on trials with an 8 s pre-cue (Figure 5O), a significant day effect, showed that there were higher percent correct responses on day 3 than day 1 (*p* = 0.047). Collectively, while there were no significant genotype effects for the responses analyzed, NonTg mice made more Total Licks than 3xTg-AD, despite 3xTg-AD making more Total Visits and initiating more Total Trials. This provides evidence that NonTg mice were more successful in accessing water than 3xTg-AD. Additionally, across days, there was a decrease in the %Premature Responses and a concurrent increase in %Correct Response for all trial pre-cue delays in both groups, highlighting that both genotypes were able to improve their performance on this RT task, demonstrating learning.

### 3.5 3xTg-AD mice are impaired on the RT task with variable 2, 4, or 8 s pre-cue and 3 s cue onset

**Figure 6:**
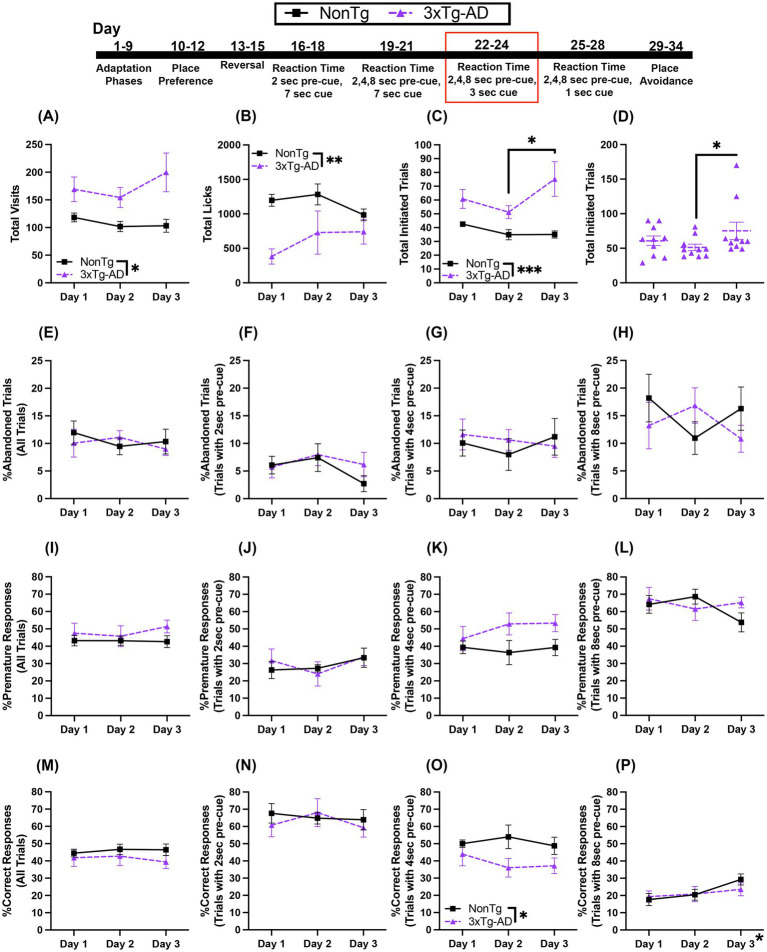
During the variable 2, 4, or 8 s pre-cue and 3 s cue duration RT task, 3xTg-AD initiated more trials but had a higher percentage of errors and a lower %Correct Responses. **(A)** 3xTg-AD made more Total Visits than NonTg, but **(B)** NonTg made more Total Licks. 3xTg-AD mice initiated more **(C)** Total Trials across days, **(D)** specifically initiated more on day 3 than day 2. The **(E)** %Abandoned Trials for all trials or the %Abandoned Trials for the 2, 4, or 8 s pre-cue **(F–H)** revealed no significant effects. **(I)** %Premature Responses for all trials was not significantly different between groups. **(J–L)** %Premature Response on trials with a 2, 4, or 8 s pre-cue delay did not differ by day or genotype. **(M)** Total %Correct Responses and the **(N)** %Correct Responses for trials with a 2 s pre-cue were similar between genotypes. **(O)** 3xTg-AD mice had a lower %Correct Response for trials with a 4 s pre-cue than NonTg. **(P)** %Correct Responses increased across days for trials with an 8 s pre-cue, but there were no genotype difference. Data are reported as means ± SEM. *p < 0.05, **p < 0.01, ***p < 0.001.

In this RT task, the first nose poke at the assigned corner initiates a trial in which the mouse must wait 2, 4, or 8 s before making a second nose poke to access water during the 3 s that the LED light cue is on. We found a significant main effect of genotype for Total Visits, where 3xTg-AD mice made more visits than NonTg mice (Figure 6A). However, for Total Licks (Figure 6B), NonTg made significantly more than 3xTg-AD mice. For Total Trials initiated (Figure 6C), we found a main effect of genotype, where 3xTg-AD mice initiated more than NonTg mice. Additionally, there was a significant day by genotype interaction (Figure 6D); 3xTg-AD mice initiated more Total Trials than NonTg on day 1 (*p* = 0.015), day 2 (*p* = 0.012), day 3 (*p* = 0.004). Further, 3xTg-AD initiated more trials on day 3 than on day 2 (*p* = 0.045).

3xTg-AD and NonTg mice did not differ in the total %Abandoned Trials, the %Abandoned Trials with 2 s pre-cue, %Abandoned Trials with 4 s pre-cue, or %Abandoned Trials with 8 s pre-cue (Figures 6E–H). There were no significant day or genotype effects for %Premature Responses for all trials or %Premature Responses for trials with 2 s pre-cue (Figures 6I,J). For %Premature Responses for trials with 4 s pre-cue, 3xTg-AD mice trended (Figure 6K) towards making more premature responses than NonTg. There were no significant main effects or interactions for %Premature Responses for trials with 8 s pre-cue (Figure 6L). For %Correct Responses for all trials and for %Correct Response for trials with 2 s pre-cue, we found no significant main effects or interactions (Figures 6M,N). However, there was a main effect of genotype for %Correct Responses on trials with 4 s pre-cue (Figure 6O), with NonTg mice having a higher %Correct Responses on trials with 4 s pre-cue than 3xTg-AD mice. The %Correct Responses on trials with 8 s pre-cue (Figure 6P), increased across days, with higher percentage of correct responses on day 3 than day 1 (*p* = 0.019), but there were no significant genotype effects or interactions.

Collectively, NonTg mice made more Total Licks than 3xTg-AD, despite 3xTg-AD having more Total Visits and initiating more Total Trials, highlighting that NonTg mice were more successful in obtaining water than 3xTg-AD. Interestingly, both genotypes had greater than 50% Correct Responses in trials with 2 s pre-cue, but less than 30% of Correct Responses in trials with 8 s pre-cue. This suggests that both genotypes were able to access water more readily when there was the short wait of a 2 s pre-cue, but they struggled with the long wait of an 8 s pre-cue, which is also evident by the increased %Premature Reponses at 8 s. However, for the 4 s pre-cue duration, there were interesting genotype differences; 3xTg-AD had lower %Correct Response than NonTg. This indicates that the 4 s pre-cue was a more difficult condition for the 3xTg-AD mice than the NonTg, but at the 8 s pre-cue delay, both genotypes found the wait time difficult.

### 3.6 3xTg-AD are impaired on the RT task with variable 2, 4, or 8 s pre-cue delay and 1 s cue onset

**Figure 7:**
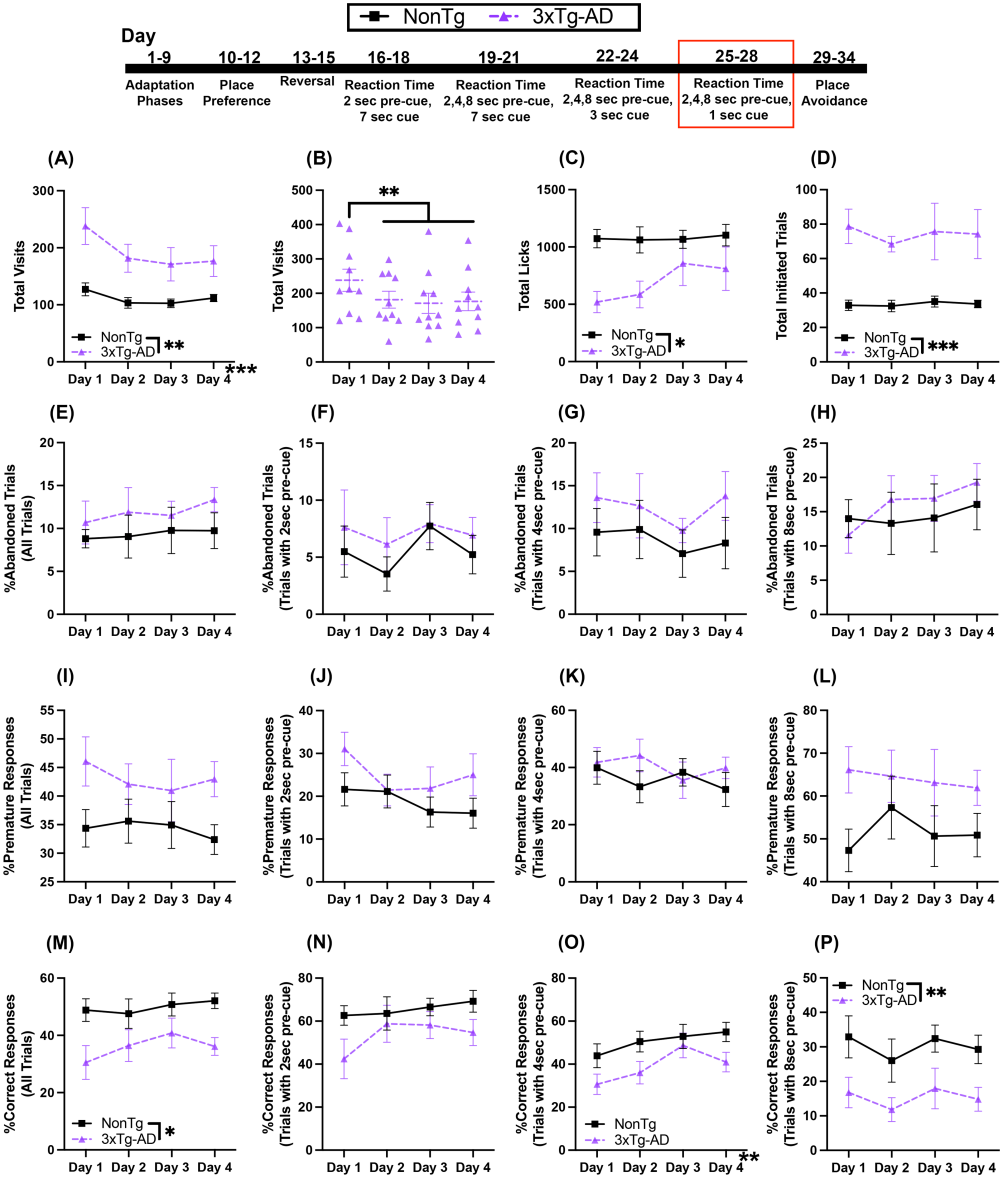
During the variable 2, 4, or 8 s pre-cue and 1 s cue duration RT task, 3xTg-AD mice initiated more trials and had a higher percentage of errors and a lower %Correct Responses. **(A,B)** 3xTg-AD made more Total Visits than NonTg and 3xTg-AD decreased Total Visits after day 1 but **(C)** NonTg made more Total Licks. 3xTg-AD initiated more **(D)** Total Trials. **(E)** %Abandoned Trials for all trials and %Abandoned Responses for 2, 4, or 8 s pre-cue **(F–H)** did not differ between genotypes. %Premature Responses for all trials **(I)** and %Premature Response on trials with 2, 4, or 8 s pre-cue **(J–L)** did not reveal any significant differences. **(M)** %Correct Responses for all trials was higher in NonTg than in 3xTg-AD mice. **(N)** The %Correct Responses for trials with 2 s pre-cue were similar for both genotypes. **(O)** %Correct Responses increased across days for trials with 4 s pre-cue. **(P)** %Correct Responses for trials with 8 s pre-cue were higher in NonTg than in 3xTg-AD mice. Data are reported as means ± SEM. *p < 0.05, **p < 0.01, ***p < 0.001.

In this RT task, the first nose poke at that corner initiates a trial in which the mouse must wait 2, 4, or 8 s before making a second nose poke to access water during the 1 s that the LED light cue is on. For Total Visits, there was a significant day effect (Figure 7A), where Total Visits decreased across days, with more Total Visits on day 1 than day 2 (*p* = 0.002), day 3 (*p* < 0.001), and day 4 (*p* = 0.006). Additionally, 3xTg-AD made more Total Visits than NonTg. There was also a significant interaction of day by genotype for Total Visits; 3xTg-AD mice made more Total Visits (Figure 7B) on day 1 (*p* = 0.003), day 2, (*p* = 0.006), day 3 (*p* = 0.030), and day 4 (*p* = 0.025) than NonTg mice. Additionally, 3xTg-AD made fewer Total Visits as days progressed with more visits on day 1 than on day 2 (*p* = 0.002), day 3 (*p* < 0.001), or day 4 (*p* = 0.002). Interestingly, while 3xTg-AD had more Total Visits, NonTg made significantly more Total Licks (Figure 7C) than 3xTg-AD. 3xTg-AD initiated more Total Trials (Figure 7D), than NonTg.

For %Abandoned Trials for all trials, %Abandoned Trials with 2 s pre-cue, %Abandoned Trials with 4 s pre-cue, and %Abandoned Trials with 8 s pre-cue, there were no significant main effects or interaction between genotypes and across days (Figures 7E–H). For %Premature Responses for all trials, %Premature Responses with 2 s pre-cue, %Premature Responses with 4 s pre-cue, and %Premature Responses with 8 s pre-cue, there were no significant main effects or interactions between genotypes or across days (Figures 7I–L). Notably, NonTg mice had higher %Correct Responses for all trials than 3xTg-AD (Figure 7M). For %Correct Response for trials with 2 s pre-cue (Figure 7N), there were no significant main effects or interactions. For %Correct Responses for trials with a 4 s pre-cue (Figure 7O), there was a significant effect of day, with a higher %Correct Responses on day 3 (*p* < 0.001) and 4 (*p* = 0.023) than on day 1. For %Correct Responses for trials with an 8 s pre-cue (Figure 7P), NonTg had a higher %Correct Response than 3xTg-AD.

Together, 3xTg-AD mice continued to have more Total Visits and Total Trials initiated than NonTg, but fewer Total Licks. Further, 3xTg-AD mice had lower %Correct Response than NonTg. Interestingly, this difference was observed in the 8 s pre-cue trials. This contrasts with the results from the previous RT task, where the 8 s pre-cue trial appeared difficult for both genotypes. This highlights that both genotypes performed equally in the 4 s pre-cue, but the 3xTg-AD mice performed worse in the 8 s pre-cue trials, likely a result of the combination of a long pre-cue delay and shortened cue onset duration of 1 s, increasing demand of both of inhibitory control and attention.

### 3.7 3xTg-AD and NonTg mice perform similarly in a learned avoidance task

**Figure 8:**
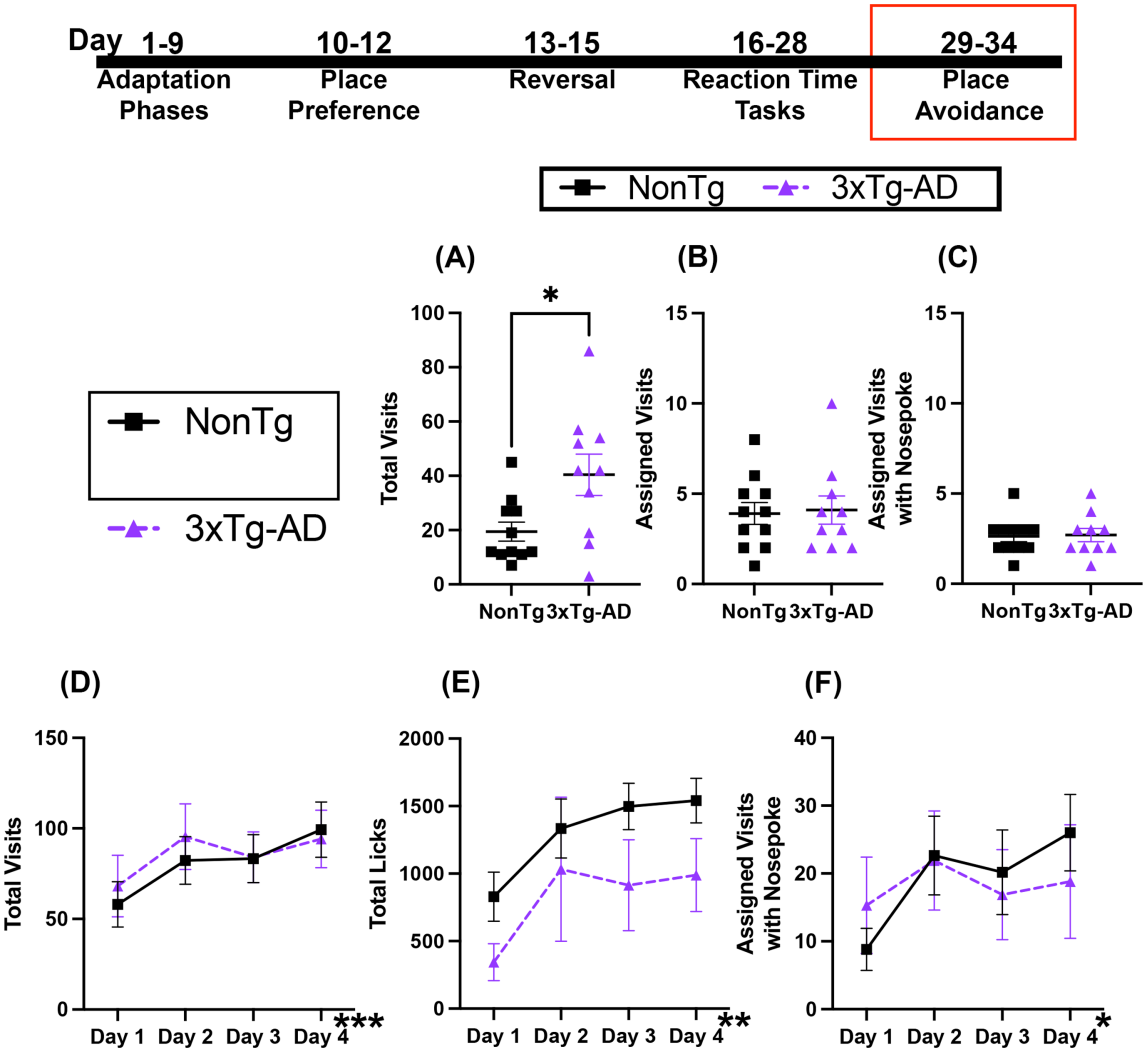
Both genotypes performed similarly during Avoidance and Extinction sessions. **(A)** 3xTg-AD mice made more Total Visits during Avoidance, but **(B)** Assigned Visits and **(C)** Assigned Visits with Nose Poke were similar and low for both groups. For the Extinction session, both groups similarly increased **(D)** Total Visits, **(E)** Total Licks, and **(F)** Total Visits with Nose Poke. Data are reported as means ± SEM. *p < 0.05, **p < 0.01, ***p < 0.001.

During the avoidance phase, mice received an air puff when they nose poked in the assigned corner, which remained the same as the RT tasks. For Total Visits (Figure 8A) to a corner, 3xTg-AD mice made more Total Visits than their NonTg counterparts. However, there were no significant main effects or interactions in Assigned Visits or Assigned Visits with a Nose Poke (Figures 8B,C), which was low in both groups, demonstrating aversion to the air puff.

Retention testing and extinction occurred 24-h following the avoidance session. Both genotypes increased their Total Visits and Licks across days (Figures 8D,E). For Total Visits, there was a significant effect of day, with more Total Visits on days 2 (*p* = 0.037) and day 4 (*p* = 0.014) than day 1. For Total Licks, there was a significant day effect, with more Total Licks on days 3 (*p* = 0.042) and 4 (*p* = 0.001) than on day 1. Further, Assigned Visits with a Nose Poke (Figure 8F) showed a significant increase across days, with more on day 2 (*p* = 0.033) and day 4 (*p* = 0.020) than on day 1. Collectively, both genotypes displayed similar avoidance of corners during the Avoidance session, suggesting similar learning for aversive events. Similarly, both groups of mice increased Total Visits, Total Licks, and Assigned Visits with a Nose Poke across days, suggesting similar extinction of adverse associations.

### 3.8 3xTg-AD mice show elevated levels of Aβ and tau pathology in the cortex

**Figure 9:**
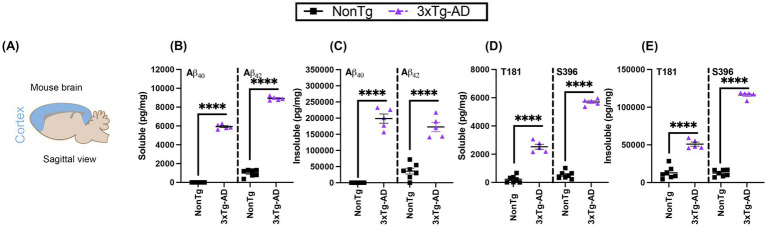
3xTg-AD mice exhibit characteristic Alzheimer’s pathology in the cortex. **(A)** Schematic representation of the brain area sampled. **(B)** Soluble Aβ40 (left) and Aβ42 (right) was elevated in 3xTg-AD mice. **(C)** Insoluble Aβ40 (left) and Aβ42 (right) was elevated in 3xTg-AD mice. **(D)** Soluble phosphorylated tau at threonine (T) 181 (left) and at serine (S) 396 (right) was elevated in 3xTg-AD. **(E)** Insoluble phosphorylated tau at T181 (left) and at S396 (right) was elevated in 3xTg-AD, but not in NonTg. Data are reported as means ± SEM. ****p < 0.0001.

Since differences in performance were seen in cortical dependent task, we confirmed the presence of AD pathology in the cortex of the 3xTg-AD mouse model to better illustrate the link between impairment in the IntelliCage and the presence of cortical pathology. We used ELISAs to measure Aβ and tau phosphorylation pathological markers in the cortex (Figure 9A). We assessed pathological isoforms of Aβ, specifically soluble and insoluble fractions of Aβ40 and 42 (Dave et al., 2023; Deture and Dickson, 2019; Judd et al., 2023). We found significantly higher levels of soluble Aβ40 (*t*(10) = 63.57, *p* < 0.0001) and Aβ42 (*t*(10) = 43.67, *p* < 0.0001) in 3xTg-AD compared to NonTg mice (Figure 9B). Additionally, insoluble levels of Aβ40 (*t*(10) = 16.73, *p* < 0.0001) and Aβ42 (*t*(10) = 8.533, *p* < 0.0001) were present and significantly elevated in the 3xTg-AD mice compared to the NonTg counterparts (Figure 9C).

Next, we assessed the levels of tau hyperphosphorylation in the cortex at pathological phosphorylation sites observed in AD (Dave et al., 2021; Judd et al., 2023; Karikari et al., 2020; Mondragón-Rodríguez et al., 2014). We specifically examined phosphorylated tau at threonine 181 (T181) and serine 396 (S396), and found significant genotype effects for both T181 (*t*(10) = 12.63, *p* < 0.0001) and S396 (*t*(10) = 34.97, *p* < 0.0001), with higher levels evident in the 3xTg-AD than NonTg (Figure 9D). We also found a significant main effect of genotype for insoluble pathological tau (Figure 9E), with 3xTg-AD showing higher levels of T181 (*t*(10) = 8.857, *p* < 0.0001) and S396 (*t*(10) = 34.97, *p* < 0.0001) than NonTg counterpart, collectivity validating the presence of humanized Aβ and tau pathogenesis in 3xTg-AD mice.

## 4 Discussion

We used the IntelliCage to implement RT tasks of increasing difficulty to determine cognitive impairments in the 3xTg-AD mouse model of AD. During the Adaptation phases, Place Preference, Reversal, Avoidance, and Extinction, both 3xTg-AD and NonTg mice were able to successfully perform the tasks, with a similar percentage of correct responses. Consistent with our previous reports, 3xTg-AD mice made more Total Visits and Total Licks in multiple phases of the experiment that likely reflected difference in motivation (Winslow et al., 2021). However, despite the higher motivation for water seeking in the 3xTg-AD, as the tasks difficulty increased, NonTg mice made more licks and had higher %Correct Responses than 3xTg-AD mice. Further, during the 3rd RT task (variable 2, 4, 8 s pre-cue with 3 s cue), genotype differences appeared for some pre-cue durations, but not others. For both genotypes, %Correct Responses were higher for trials with a 2 s pre-cue and lower for trials with an 8 s pre-cue. This suggests that waiting during the 2 s pre-cue was an easy demand for both genotypes, but the 8 s pre-cue taxed the impulse control of both genotypes. Interestingly, differences emerged on trials with a 4 s pre-cue, where 3xTg-AD had a lower %Correct Responses than NonTg mice, suggesting that impulse control differences are seen at this pre-cue duration with a 3 s cue duration. Further, on the final RT task (variable 2, 4, or 8 s pre-cue with 1 s cue), NonTg mice had higher %Correct Response at the 8 s pre-cue than 3xTg-AD mice, despite having struggled with this pre-cue on the previous RT task. This suggests that NonTg mice were able to learn the rule of the longer pre-cue delay, illustrating impulsivity control, but 3xTg-AD continued to show impairments with this wait period, which also increased demand in attention given the short 1 s cue onset duration. Notably, 3xTg-AD have been shown to exhibit impaired attention and heightened impulsivity in the 5-choice serial reaction time task (5-CSRTT) (Romberg et al., 2011). This shows that the variable cued RT task in the IntelliCage can be a useful tool for investigating AD-related cognitive impairments in the 3xTg-AD mouse and how error types can differ as cognitive load changes.

In addition to progressively harder RT task challenges, we also implemented other changes from our previous work to aide in detecting genotype differences. First, mice were water restricted and only able to access water during 3-h of the active (dark) part of the light cycle. This was done for the goal of equalizing water seeking motivation between the genotypes. This has been done previously in other reports in other models of AD (Masuda et al., 2016, 2018) and is commonly employed in operant tasks (Goltstein et al., 2018). We still observed higher motivation to drink in the 3xTg-AD than in the NonTg, as reflected in the greater number of Total Visits and Total Trials initiated, but in the metrics that reflect a successful trial, NonTg performed better, including more Total Licks and higher %Correct Response during RT tasks. Second, compared to our previous report, mice were given more opportunities to engage in the tasks after a failure to perform. In our previous report (Winslow et al., 2021), mice were removed from the task if they failed to acquire water after two 24-h periods without consuming water, resulting in a 43% failure rate for 3xTg-AD mice. Here, we returned mice to the IntelliCage after a failure to drink water until the end of the Reversal task. Consequently, most mice with one failure to drink in a 48-h period were able to complete the task and the only mice that were removed from RT tasks were two NonTg mice that failed to drink in both PP and Reversal PP. This resulted in a low dropout rate and a greater range of performance. This prevented biased interpretation issues that could occur from collecting data from only the best performers, instead of a broader range of performances.

The current results with progressively difficult tasks parallel well established behavioral tests that have increasing difficulty, including the win-shift form of the Radial Arm Maze (RAM) and the 5-CSRTT, but with the exception of being tested in a social environment in the animals’ home cage without experimenter disruption. In the win-shift RAM paradigm (Bernaud et al., 2022; Clark et al., 2015; Hyde et al., 1998; Koebele et al., 2020), half of the maze arms are bated (food for the land version or escape platform for water version). As the rodent finds the bated arm, there are fewer possible correct responses. This increases memory load across trials in a day; the animal must remember which arms are bated and which arms to not revisit because they are no longer bated. Like the RT tasks presented in the current study, the win-shift version of the RAM has various types of possible errors that can indicate impairments in various memory domains, such as working memory. While most animals can find a platform on easier trials, differences are often observed when working memory load is higher (Bernaud et al., 2022; Hyde et al., 1998). In the 5-CSRTT, attention is assessed by detecting a brief visual stimulus (such as a LED) presented pseudorandomly, across several spatial locations in a five-nose port box, and mice have to nose poke at the signaled port to get a reward (Asinof and Paine, 2014; Sanchez-Roige et al., 2012). Response errors are more likely to occur under more restrictive conditions or with a long delay before being able to respond (Asinof and Paine, 2014; Sanchez-Roige et al., 2012). The outcomes of the more challenging trials of win-shift RAM and 5-CSRTT parallel the results seen here, both genotypes could successfully complete the easier RT sessions and RT trials with a 2 s pre-cue, but differences emerged as difficulty increased or required a longer delay before responding. The 3xTg-AD mice struggled, but the NonTg mice had higher %Correct Response on the 4 s pre-cue trials in the 3^rd^ RT task and on the 8 s pre-cue trial on the 4^th^ RT task. This suggests that using RT paradigms in the IntelliCage can allow experimenters to perform a nuanced error breakdown. Thus, we propose that the IntelliCage has the potential for assessing complex behavioral patterns in AD mouse models and can be interpreted within the framework established in RAM and 5-CSRTT analysis, with the advantage of the allowing mice to be tested in a social home environment, and not needing to be removed from their home cage and handled by experimenters, allowing for less stress while probing behavior.

Many traditional behavioral testing paradigms assess hippocampal dependent behavior, where impairments are seen in the 3xTg-AD mouse compared to NonTg at this age (11.5 months) (Dave et al., 2023; Parachikova et al., 2010; Roda et al., 2020). However, for the hippocampal dependent tasks in the IntelliCage, Place Preference (Ferbinteanu and McDonald, 2001) and aversive conditioning (Lee and Kesner, 2004), 3xTg-AD and NonTg mice performed similarly. An important difference is that traditional water maze testing is mildly aversive, triggering a stress response in the subjects. Cognitive performance with stress follows an inverted U-shaped pattern (Salehi et al., 2010), where the right amount of stress improves performance, but too much stress is impairing. Given that 3xTg-AD mice frequently display higher anxiety-like behavior and stress reactivity (Blázquez et al., 2014), it is possible that they find traditional water maze testing more stressful than NonTg and this could impact the interpretations of their spatial memory. However, it is noteworthy to consider the value of these tasks for an IntelliCage paradigm in the 3xTg-AD mouse model. For several reasons, we propose that they have value in an IntelliCage behavioral battery. First, since all mice were able to complete these tasks, they act as a “probe task” and illustrate that subjects can learn the rules of the task. Second, the present study used mice that were aged, but were not treated with disease altering variables. In a study where pathology is manipulated to unveil mechanism, treated for therapeutic purposes, or mice are aged to more advanced stages of pathology, these easier tasks may show genotype performance differences. Third, preserving PP and Reversal prior to RT provides time for animals to learn the basic rules of the task. Additionally, by returning mice to the IntelliCage after failing to access water up until the RT task, we were able to maintain high subject numbers throughout testing and have lower attrition than in our previous studies, with only two NonTg mice being excluded after failure to engage in PP and Reversal. Thus, all tasks are critical for successful implementation of more complex task parameters, such as those implemented in the RT task.

While hippocampal dependent processes are often assessed in pre-clinical AD studies, other realms of behavior are also negatively impacted in AD. Indeed, impulsivity and attention deficits are commonly observed in AD patients (Bidzan et al., 2012; Keszycki et al., 2019; Sakurai et al., 2020). In AD patients, higher levels of impulsivity correspond with reduced cognitive functioning (Bidzan et al., 2012; Sakurai et al., 2020). Further, impulsivity issues are not impacted by common AD treatments and can be particularly difficult for caregivers to handle (Keszycki et al., 2019). AD patients also suffer from impaired attention (Malhotra, 2019; Rizzo et al., 2000). Like impulsivity, attentional issues correspond with worse cognitive outcomes (Rizzo et al., 2000). Here, the 3xTg-AD mice had more premature responds and fewer correct responses during multiple phases of the RT tasks, indicating impulsivity and attentional issues compared to NonTg. Further, given the relationship of impulsivity and attention issues with cognitive decline in AD patients, when 3xTg-AD mice display heighten impulsivity in the IntelliCage it can be inferred that there is greater cognitive impairment. This suggests that RT testing in the IntelliCage may be particularly useful to assess if treatments help attenuate behavioral issues.

In summary, this work shows that 3xTg-AD mice can be assessed in demanding RT tasks, increasing impulsivity control and attention load, in the IntelliCage. This work will inform the neurodegenerative field on important factors to consider when testing 3xTg-AD in the automated IntelliCage system and how modifying pre-cue and cue parameters can allow for detection of impairments.

## Data Availability

The raw data supporting the conclusions of this article will be made available by the authors, without undue reservation.
